# Reduced interleukin-6 stress reactivity in male physicians with occupational burnout

**DOI:** 10.1007/s00702-025-02945-9

**Published:** 2025-05-26

**Authors:** Christoph Woolley, Mary Princip, Claudia Hackl-Zuccarella, Bianca Auschra, Sarah Andrea Holzgang, Aju Pazhenkottil, Diego Gomez Vieito, Roland von Känel

**Affiliations:** 1 Department of Consultation-Liaison Psychiatry and Psychosomatic Medicine, University Hospital Zurich, University of Zurich, Haldenbachstrasse 16/18, 8091 Zurich, Switzerland; 2 Department of Cardiology, University Hospital Zurich, Zurich, Switzerland; 3 Cardiac Imaging, Department of Nuclear Medicine, University Hospital Zurich, Zurich, Switzerland; 4 Institute of Molecular Cancer Research, University of Zurich, Zurich, Switzerland

**Keywords:** Burnout, Interleukin-6, Cardiovascular health, Cytokine, Stress, Physician Health

## Abstract

Occupational burnout is associated with an elevated risk of cardiovascular events, but the mechanisms underlying this connection have rarely been studied. Low grade inflammation with interleukin (IL)-6 as a key player has been postulated as a possible link between low-grade inflammation and cardiovascular events. Another possibility could be attenuated inflammatory responses to stress reflecting a dysregulated innate immune response. The aim of this study was to examine the relationship between burnout and IL-6 reactivity to acute stress in physicians with burnout. Sixty male physicians were recruited, 30 with burnout, assessed with the Maslach Burnout Inventory, and 30 without burnout. Participants underwent the Trier Social Stress Test, inflicting uncontrollability, and social evaluative threat. Blood samples for IL-6 measurements were taken five times at predefined intervals during stress. Repeated measures analysis of covariance was conducted across the five time points, adjusting for age. IL-6 reactivity over time was significantly lower in physicians with burnout compared to their matched healthy counterparts (F(1.90, 108.10) = 4.88, p = 0.010) during stress. Confirming these results, there was also a significantly lower area under the curve with respect to increase in physicians with burnout compared to those without burnout (F(1, 57) = 4.99, p = 0.029). This study suggests that burnout is associated with lowered IL-6 reactivity in male physicians with burnout. An attenuated innate immune response in burnout complies with increased allostatic load such that dysfunctional stress responses might increase the risk of cardiovascular events in the long term.

## Introduction

Burnout was firstly described by Freudenberger in 1994 (Freudenberger 1994) who referred to it as a phenomenon of chronic work-related stress and high demands in helping professions. In the current scientific conceptualization, occupational burnout is seen as a multidimensional, dynamic process of chronic stress, characterized by emotional exhaustion, depersonalization and reduced personal accomplishment (Schaufeli and Bram. 2004). In accordance with a position paper by the German Association for Psychiatry and Psychotherapy (Berger et al. 2012), burnout should be described as a “risk state” caused by prolonged exposure to job stress that can arise from an imbalance between high efforts spent at work and low rewards received (Tang et al. 2018). This risk state may possibly lead to mental disorders, including depression, and physical diseases, including cardiovascular disease (CVD). This also means that psychiatric disorders, foremost depression, although showing a growing prevalence with increasing severity of burnout, should be distinguished from burnout (Berger et al. 2012).

Physicians show a two-fold higher prevalence of burnout compared to the general working population with different degrees of prevalence, depending on the specialty (Arigoni et al. 2009). In a recent systematic review including 109,628 individuals of 45 different countries, an estimated overall burnout prevalence of 67.0% in physicians has been shown. However, this review also found high heterogeneity in study designs and methods used when measuring burnout (Rotenstein et al. 2018). In a longitudinal study, the prevalence of burnout symptoms among physicians increased from 45.5% in 2011 to 54.4% in 2014 (Shanefelt et al. 2015). A study conducted in 2002 among Swiss primary care practitioners showed that 32% were considered to have a moderate degree and 4% a high degree of burnout (Goehring et al. 2005).

There is an intriguing line of research suggesting that burnout is predictive of CVD morbidity and all-cause mortality (Appels and Schouten 1991; Melamed et al. 1992; Kitaoka-Higashiguchi et al. 2009; Toker et al. 2012) with inflammatory changes as one potentially underlying mechanism (Toker et al. 2005; Känel et al. 2008). One potential specific link between burnout and CVD is interleukin-6 (IL-6), a pleiotropic cytokine, known for its function in both pro- and anti-inflammatory processes. IL-6 plays a pivotal role in various aspects of the innate immune response, including the expression of acute inflammation proteins, lymphocyte proliferation and differentiation, and the induction of homeostatic processes. IL-6 is emitted by numerous immune and non-immune cells, such as T-cells, B-cells, monocytes, fibroblasts, adipocytes, muscle cells, endothelial cells, injured myocytes, and others (Pedersen and Febbraio 2007, 2008; Kishimoto et al. 1995; Chalaris et al. 2011; Garbers et al. 2011; Uciechowski and Dempke 2020; Jones et al. 2011; Scheller et al. 2011; Starkie et al. 2003; Kanda and Takahashi 2004). Increased circulating levels of IL-6 have been shown to predict the risk of incident cardiovascular events (Papadopoulos et al. 2022; Niu et al. 2012) and are a possible target of pharmaceutical intervention in CVD prevention (Ridker and Rane 2021). Whereas the relationship between subtle increases in IL-6, indicative of low-grade inflammation, and atherosclerotic risk is well-established, initial evidence also suggests that changes in IL-6 reactivity to acute challenges may be linked to CVD risk. For instance, in perimenopausal women, heightened IL-6 reactivity to acute psychosocial stress was associated with diminished endothelium-dependent vascular function at 12 months follow-up (Zannas et al. 2020). Conversely, an attenuated feedback response on the hypothalamic-pituitary adrenal axis resulting from lower IL-6 increase during acute psychosocial stress, has been associated with an increased release of cortisol in middle-aged men (Känel et al. 2006). This, in turn, has been associated with the progression of coronary artery calcification in healthy men and women (Hamer et al. 2012). Hence, considering the pleiotropic effects of IL-6, cardiovascular consequences stemming from both excessively low and high stress reactivity in IL-6 may accumulate over time, potentially leading to overt CVD.

However, to our knowledge, there is currently no existing literature examining the relationship between burnout and IL-6 reactivity to acute psychosocial stress. This study aimed to test the hypothesis that IL-6 reactivity is perturbed in physicians with burnout when compared to their healthy counterparts.

## Materials and methods

### Participants and recruitment

The research received approval from the local ethics committee of the state of Zurich, Switzerland (BASEC-Nr. 2018-1974), and all participants, having been informed about the study procedure, provided written consent. Data collection took place from September 2019 to December 2021. Recruitment of male physicians in Switzerland was achieved through multiple approaches, including hospitals, clinics and direct outreach as part of a study aimed at gaining deeper insights into the health implications of occupational burnout on the cardiovascular system.

Comprehensive information and the study’s objectives were conveyed to interested physicians through flyers and text messages, which included contact details for inquiries. The study population comprised 60 male physicians who enrolled in the research project “Effect of burnout on myocardial blood flow: a study to assess cardiovascular health in physician burnout”. The burnout group consisted of 30 physicians with burnout, yet without clinically relevant depressive symptoms. The control group included 30 healthy physicians who were devoid of burnout and clinically relevant depressive symptoms to be included in the study, physicians had to be male, non-smokers for at least five years prior to our study, and between 28 and 65 years of age. This age range was chosen as it reflects the typical career span of physicians in Switzerland, where medical practice generally begins around age 28 and formal retirement occurs at 65. Written informed consent was obtained from all participants. For the healthy control group, low scores were required in the Maslach Burnout Inventory (MBI) (< 16 in the subscale “Emotional Exhaustion” (EE) and < 6 in the subscale “Depersonalization” (DP)) and Patient Health Questionnaire (PHQ)−9 (≤ 10 for depressive symptoms). Physicians with high scores in MBI (≥ 27 for EE, ≥ 10 DP (with EE ≥ 20)) (Goehring et al. 2005) and PHQ-9 (≤ 14 for depressive symptoms) were included in the burnout group.

Excluded were individuals with pre-existing conditions such as a familial history of hypercholesteremia, diabetes I or II, stage II hypertension (systolic BP > 160 mmHg and/or diastolic BP > 100 mmHg) or (a) known renal insufficiency with a glomerular filtration rate < 60 ml/min/1.73 m^2^. Individuals with a BMI of ≥ 35 kg/m^2^, chronic risky alcohol consumption defined as ≥ 4 standard drinks per day, taking medications that could interfere with biomarker levels (including corticosteroids, anticoagulants) were also excluded. Participants had to have no known allergies against iodinated contrast medium and no known contraindication for adenosine, betablocker or nitrates. Any known active serious disease (verified by a semi-structured history) or the decision to exercise the right to forgo information on clinically relevant cardiac imaging findings resulted in exclusion. Furthermore, participants were required to have no known history of depression or burnout in the past.

### Study procedure

After cardiac imaging in the Nuclear Medicine Department of the University Hospital of Zurich, between 7:10 am and 9:10 am, physicians were accompanied to the Stress and Behaviour Research Lab in the Department of Consultation-Liaison Psychiatry and Psychosomatic Medicine. After a light standardized breakfast, consisting of two bread rolls, an apple and water or decaffeinated tea, participants were connected to either the Finapres^®^ Nova (Finapres Medical System, Enschende, The Netherlands) or the Omron Evolv device (Omron Healthcare Co., Kyoto, Japan) for monitoring heart rate (HR) and blood pressure (BP) before, during and after the Trier Social Stress Test (TSST). The documentation of hemodynamic data had to be adapted after 13 participants, due to technical issues with the Finapres^®^ Nova, requiring the switch to the Omron Evolv device. Before, during and after the TSST, during predetermined setpoints, blood samples were collected by trained medical personnel using an intravenous 18-gauge catheter, previously inserted during cardiac imaging into an antecubital vein.

### Trier social stress test

The TSST was applied (Maslach et al. 1996) to assess stress reactivity in both groups and typically lasted approximately 15 min with three 5-min parts. A panel of three judges (trained staff), equipped with audio recorder and video camera, instructed each participant to prepare a 5-min presentation for a simulated job interview. To prepare the presentation, the participant could use paper and pen, but had to hand out his notes at the beginning of the presentation. During the 5-min presentation, the judges observed the participant without giving any comments or facial expression. The participant was asked to continue if he would stop before the entire 5 min ran out. The presentation was then immediately followed by a mental arithmetic component, during which the participant was asked to count backwards from 2043 in steps of 17. If the participant made a mistake, he was asked to start again from the beginning. This component lasted for 5 min as well and was followed by a recovery period of 90 min. After the recovery phase, the participant got informed, that the purpose of the test was to create stress, and that the results do not reflect personal abilities. 10 min before the TSST, immediately after the TSST, and three times during the recovery phase, i.e., 15 min, 45 min and 90 min after cessation of the TSST, blood samples were collected to evaluate changes in circulating IL-6 levels. Heart rate and blood pressure were measured 10 min before the TSST and immediately after the TSST to verify that the stress task was effective in eliciting a stress response (manipulation check).

### Psychometric assessment

To assess the severity of burnout, the German version of the Maslach Burnout Inventory-Human Services Survey (MBI-HSS) was used (Papadopoulos et al. 2022; Niu et al. 2012). This instrument is a self-administered questionnaire that was designed to assess the three subscales of burnout: “Emotional Exhaustion (EE) (9 items), “Depersonalization” (DP) (5 items), and “Personal Accomplishment” (PA) (5 items). All items are scored using a 7-point frequency scale ranging from 0 ("never") to 6 ("daily"). Items are written in the form of statements about personal feelings or behaviours (e.g. “I feel burned out from my work”). Each subscale is considered separately. The items in the EE dimension assess feelings of emotional overextension and exhaustion. The DP subscale measures an unfeeling and impersonal response toward recipients of care. The PA subscale evaluates feelings of competence and successful achievement at work. The subscale EE showed good internal consistency (Cronbach’s α = 0.824), the subscale of PA showed excellent internal correlation (Cronbach’s α = 0.940) and the subscale DP showed sufficient internal correlation (Cronbach’s α = 0.762).

To assess depressive symptoms, we applied the German version of the PHQ-9. The PHQ-9 is a self-administered questionnaire consisting of 9 questions to screen for possible depression. All items are scored using a 4-point frequency scale from"never"to"daily". There are score ranges that define “low,” “moderate” and “high” levels of each scale based on the 0–3 scoring. Values between 20 and 27 indicate severe depressive symptomatology, values between 15 and 19 moderately severe symptoms, 10–14 moderate symptoms, 5–9 mild symptoms and 0–4 none to minimal symptoms. In general, a total score of 10 or above is suggestive of the presence of clinically relevant depressive symptoms (Ridker and Rane 2021). The PHQ-9 has shown a particularly good internal consistency (Cronbach’s α = 0.891).

### Biochemical analyses

#### Interleukin-6 levels

For the quantification of IL-6 venous blood was drawn in EDTA-coated monovettes (Sarstedt, Nuembrecht, Germany) and immediately centrifuged for 10 min at 2000G and room temperature. Obtained plasma was stored at – 80 °C until analysis. Plasma cytokine level of IL-6 was determined with a high sensitivity sandwich immunoassay (IBL International, Hamburg, Germany).

### Data analysis

Data were analysed with IBM SPSS Statistics for Windows, Version 29.0 (Armonk, NY: IBM Corp). The level of significance was set at p ≤ 0.05 for all calculations (two-tailed). Missing data was replaced by the expectation–maximization-algorithm. Interleukin-6 levels showed a normal distribution by visual inspection. We used a repeated measures ANOVA with adjustment for age to compare IL-6 values across the five measurement points between the two groups. As a confirmatory analysis and to more precisely compare the reactivity of IL-6 concentration between groups, we calculated the area under the curve with respect to increase. This calculation is based on a trapezoid formula frequently employed in psychoneuroendocrinology research to estimate changes in biological measures over time when utilizing repeated measurements (Pruessner et al. 2003). We used independent‐sample t‐test to determine group differences in area under the curve with respect to increase. One participant had no MBI and PHQ-9 data on the examination day; instead, we used the corresponding data collected during the telephone interview.

## Results

### Sample characteristics

Table 1 shows the characteristics of the 60 male physicians, 30 in the burnout group and 30 in the control group without burnout. Compared to the control group, physicians with burnout were significantly younger, had greater job stress, worked more hours per week and had more severe depressive symptoms.

**Table 1:** Characteristics of the sixty study participants

Variable	Burnout (n = 30) M (SD)	Control (n = 30) M (SD)	p value
Working hours per week	57.35 (8.99)	54.72 (11.67)	0.166
Age, years	46.77 (10.56)	52.93 (7.48)	0.012
Emotional exhaustion, score	29.12 (7.13)	6.67 (3.99)	< 0.001
Depersonalization, score	11.33 (7.00)	3.07 (3.60)	< 0.001
Personal accomplishments, score	12.03 (6.74)	5.67 (4.37)	< 0.001
PHQ-9_Sumscore	10.36 (2.65)	3.06 (2.96)	< 0.001

The reported values represent the mean with the standard deviation being in parentheses. Independent samples t-tests were utilized to compare the means of the variables between the burnout and control group

*PHQ-9* Patient Health Questionnaire-9

### Manipulation check

There was a significant stress-induced increase (all p values < 0.001) in heart rate (70.1 bpm vs. 80.8 bpm), systolic blood pressure (128.9 mmHg vs. 144.8 mmHg) and diastolic blood pressure (81.2 mmHg vs. 90.6 mmHg) across all study participants, verifying that the TSST elicited a significant hemodynamic response. The burnout and control group did not significantly differ in stress-induced increases in hemodynamic measures, with adjustment for age and the type of hemodynamic monitor used.

### IL-6 response to stress

A repeated measures ANOVA with Greenhouse–Geisser correction for the degrees of freedom was used for violations of sphericity (Mauchly-test of sphericity p ≤ 0.001), showing significant group differences (F(1.89, 110.02) = 4.64, p = 0.012), persistent when adjusting for age (F(1.90, 108.10) = 4.88, p = 0.010), in physicians with burnout compared to their healthy counterparts.

There were also significant differences (F(1, 58) = 5.32, p = 0.025 in IL-6 area under the curve with respect to increase with physicians with burnout showing lower IL-6 reactivity compared to the non-burnout group, even when adjusted for age (F(1, 57) = 4.99, p = 0.029).

**Fig. 1:**
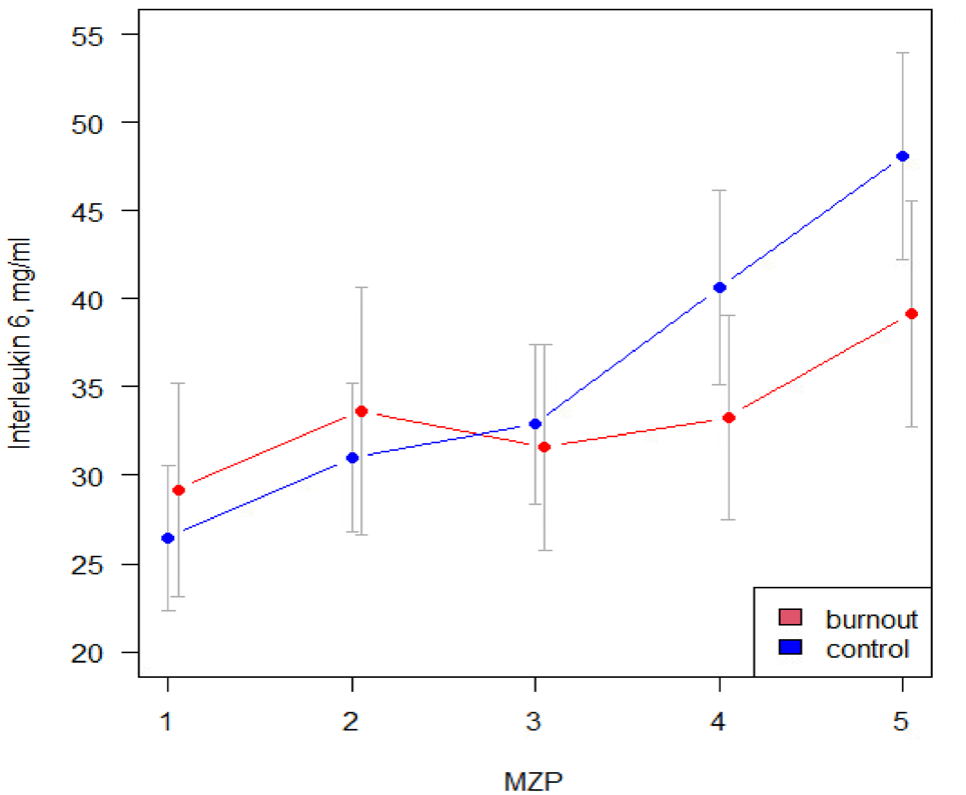
Interleukin-6 reactivity to TSST in burnout group and control group

Figure 1 shows the mean IL-6 concentration of both groups during the measurement period.

## Discussion

Our results show statistically significant reduced IL-6 reactivity in physicians with burnout compared to the non-burnout group. As of today, there is no literature available that directly compares IL-6 reactivity in individuals with burnout to that of controls. Elevated IL-6 levels have been shown to be associated with an increased risk of adverse cardiovascular events (Papadopoulos et al. 2022; Niu et al. 2012; Ridker and Rane 2021). We found no statistically significant differences in IL-6 levels between the two groups at baseline. The emphasis of this comparison lies on the reactivity of IL-6 levels. Research conducted by Pedersen and Febbraio (2007, 2008) has suggested that acute changes in IL-6 levels following stress, although in the context of physical stress, may have advantageous effects on acute insulin sensitivity and glucose homeostasis. These findings suggest potential benefits in terms of reducing cardiovascular risk factors in male physicians.

A more recent systematic review by Marsland et al. (2017) compared the effect of a defined social stressor across 27 studies and demonstrated significant changes in circulating IL-6 levels between 10 to 120 min after stress exposure. However, individuals with burnout seem to exhibit a diminished response compared to their healthy counterparts. The impaired IL-6 response observed in individuals with burnout suggests a possible link between burnout and CVD, where the weakened post-stress IL-6 response may attenuate the potential short-term beneficial effects of IL-6. Although speculative, we propose three explanations for this intriguing observation. First, in the context of ongoing job stress, the immune system may become less responsive or dysregulated in its reaction to acute stressors. This possibility could be explored further through longitudinal studies. Second, the physical and emotional exhaustion of individuals with burnout could extend to the immune system, where resources needed for an acute inflammatory response, like IL-6 production, may be depleted or less readily available. Third, dysregulation of the hypothalamic-pituitary adrenal axis can affect the acute release of cortisol, which, in turn, can influence the production of IL-6. Further research is necessary to substantiate these assumptions and unravel the intricate relationship between burnout, the acute stress response, and IL-6 production.

## Strengths and limitations

One of the primary strengths of this study is the homogeneity of its participants, as all individuals involved white males with similar socio-economic and educational background. This homogeneity eliminates many potential confounding variables, a quality that is further reinforced by the rigorous selection process, which excluded individuals with certain potential confounding factors. Additionally, the use of the TSST for comparing IL-6 changes post-stress (Marsland et al. 2017) is noteworthy, as it also yielded significant results in our study.

While the homogeneity of the study’s participants stands as one of its notable strengths, it also represents one of its significant weaknesses. The relatively small sample size limited statistical power and the stringent inclusion criteria resulted in a highly selective cohort, limiting generalizability. Specifically, the study included only 60 male physicians, which limits the generalizability of the findings both to the broader healthcare workforce, including female physicians, and to the wider population of male physicians, given the specific and narrowly defined sample. The restricted age range of 28–65 years, although chosen to reflect the typical professional lifespan of physicians in Switzerland, may further limit the applicability of the results to younger or older individuals in the healthcare workforce outside this range. The study did not include a comprehensive assessment of other potentially relevant psychosocial variables, such as work-life balance, perceived job control, or job satisfaction, which may influence or mediate the observed relationship between burnout and IL-6 stress reactivity. This omission limits the depth of interpretation and may have obscured additional explanatory pathways. Finally, although the instruments used for assessing burnout and depressive symptoms are validated and widely applied, reliance on self-report measures remains a potential source of bias. Future studies may benefit from complementing questionnaire-based assessments with clinician-administered semi-structured interviews to improve diagnostic precision.

## Conclusions

Burnout appears to influence an individual’s ability to mount and adequate physiological response to stress. A possible link between burnout and elevated risk of CVD could be through the disruption of the acute stress reactivity, not through permanent low-grade inflammation as in obesity or old age. While our findings suggest that burnout may represent a physiological risk state with potential implications for cardiovascular health, these conclusions should be interpreted with caution given the limitations of this study. Burnout and its consequences, not only on the cardiovascular and overall health of physicians and other medical professionals, but also on the healthcare system, warrant serious attention. Given a pivotal role of the pleiotropic cytokine IL-6 in health and disease, efforts to detect burnout early and better understand its physiological and psychological impact remain essential for effective prevention and intervention. Future studies should examine whether remission of burnout is accompanied by normalization of IL-6 stress responses, which could further underscore inflammatory changes as a potential psychobiological mechanism linking burnout and cardiovascular risk.

## References

[CR1] Appels A (2004) Exhaustion and coronary heart disease: the history of a scientific quest. Patient Educ Couns 55(2):223–229 15530758 10.1016/j.pec.2003.09.008

[CR2] Appels A, Schouten E (1991) Burnout as a risk factor for coronary heart disease. Behav Med 17:53–59 1878609 10.1080/08964289.1991.9935158

[CR3] Arigoni F, Bovier PA, Mermillod B, Waltz P, Sappino AP (2009) Prevalence of burnout among Swiss cancer clinicians, paediatricians and general practitioners: who are most at risk? Support Care Cancer 17:75–81 18528715 10.1007/s00520-008-0465-6

[CR4] Berger M, Linden M, Schramm E, Hillert A, Voderholzer U, Maier W (2012) Positionspapier der Deutsche Gesellschaft für psychiatrie: psychotherapie und nervenheilkunde (DGPPN) zum thema burnout. Nervenarzt 4:537–543

[CR5] Büssing A, Perrar KM (1992) Die Messung von burnout: Untersuchung einer deutschen Fassung des Maslach burnout inventory (MBI-D) [Measuring burnout: a study of a German version of the Maslach burnout inventory (MBI-D)]. Diagnostica 38(4):328–353 8004334

[CR6] Chalaris A, Garbers C, Rabe B, Rose-John S, Scheller J (2011) The soluble interleukin 6 receptor: generation and role in inflammation and cancer. Eur J Cell Biol 90:484–494 21145125 10.1016/j.ejcb.2010.10.007

[CR7] Cohen R, Bavishi C, Haider S, Thankachen J, Rozanski A (2017) Meta-analysis of relation of vital exhaustion to cardiovascular disease events. Am J Cardiol 119(8):1211–1216 28215416 10.1016/j.amjcard.2017.01.009

[CR8] Freudenberger HJ (1994) Staff burn-out. J Soc Issues 30(1):159–165

[CR9] Gajewski PD, Boden S, Freude G, Potter GG, Claus M, Bröde P, Watzl C, Getzmann S, Falkenstein M (2017) Executive control, ERP and pro-inflammatory activity in emotionally exhausted middle-aged employees: comparison between subclinical burnout and mild to moderate depression. Psychoneuroendocrinology 86:176–186 28972891 10.1016/j.psyneuen.2017.09.017

[CR10] Garbers C, Thaiss W, Jones G, Waetzig G, Lorenzen I, Guilhot F et al (2011) Inhibition of classic signaling is a novel function of soluble glycoprotein 130 (sgp130), which is controlled by the ratio of interleukin 6 and soluble interleukin 6 receptor. J Biol Chem 286:42959–42970 21990364 10.1074/jbc.M111.295758 PMC3234812

[CR11] Goehring C, Bouvier Gallacchi M, Kunzi B, Bovier P (2005) Psychosocial and professional characteristics of burnout in Swiss primary care practitioners: a cross-sectional survey. Swiss Med Wkly 135:101–108 15832226 10.4414/smw.2005.10841

[CR12] Hamer M, Endrighi R, Venuraju SM, Lahiri A, Steptoe A (2012) Cortisol responses to mental stress and the progression of coronary artery calcification in healthy men and women. PLoS ONE 7(2):e31356. 10.1371/journal.pone.0031356. (**PMID: 22328931; PMCID: PMC3273460**) 22328931 10.1371/journal.pone.0031356 PMC3273460

[CR13] Hartman J, Frishman WH (2014) Inflammation and atherosclerosis. Cardiol Rev 22(3):147–151. 10.1097/crd.0000000000000021 24618929 10.1097/CRD.0000000000000021

[CR14] Honkonen T, Ahola K, Pertovaara M, Isometsa E, Kalimo R, Nykyri E, Aromaa A, Lonnqvist J (2006) The association between burnout and physical illness in the general population: results from the Finnish Health 2000 Study. J Psychosom Res 61:59–66 16813846 10.1016/j.jpsychores.2005.10.002

[CR15] ICD-10 (1992) ICD-10 Classifications of Mental and Behavioural Disorder: Clinical Descriptions and Diagnostic Guidelines. World Health Organisation, Geneva

[CR16] IL6R Genetics Consortium Emerging Risk Factors Collaboration (2012) Interleukin-6 receptor pathways in coronary heart disease: a collaborative meta-analysis of 82 studies. The Lancet 379(98):1205–1213. 10.1016/s0140-6736(11)61931-4 10.1016/S0140-6736(11)61931-4 PMC3316940 22421339

[CR17] Jones SA, Scheller J, Rose-John S (2011) Therapeutic strategies for the clinical blockade of IL-6/gp130 signaling. J Clin Invest 121(9):3375–3383. 10.1172/JCI57158. (**PMID: 21881215; PMCID: PMC3163962**) 21881215 10.1172/JCI57158 PMC3163962

[CR18] Jonsdottir IH, Dahlman AS (2019) Mechanisms in endocrinology: Endocrine and immunological aspects of burnout: a narrative review. Eur J Endocrinol 180(3):R147–R158. 10.1530/EJE-18-0741 30576285 10.1530/EJE-18-0741 PMC6365671

[CR19] Kanda T, Takahashi T (2004) Interleukin-6 and cardiovascular diseases. Jpn Heart J 45(2):183–193. 10.1536/jhj.45.183 15090695 10.1536/jhj.45.183

[CR20] Kidd T, Carvalho LA, Steptoe A (2014) The relationship between cortisol responses to laboratory stress and cortisol profiles in daily life. Biol Psychol 99:34–40 24582772 10.1016/j.biopsycho.2014.02.010 PMC4031630

[CR21] Kirschbaum C, Pirke KM, Hellhammer DH (1993) The “Trier Social Stress Test”: a tool for investigating psychobiological stress responses in a laboratory setting. Neuropsychobiology 28(1–2):76–81 8255414 10.1159/000119004

[CR22] Kishimoto T, Akira S, Narazaki M, Taga T (1995) Interleukin-6 family of cytokines and gp130. Blood 86(4):1243–1254. 10.1182/blood.V86.4.1243.bloodjournal8641243. (**ISSN 0006-4971**) 7632928

[CR23] Kitaoka-Higashiguchi K, Morikawa Y, Miura K, Sakurai M, Ishizaki M, Kido T, Naruse Y, Nakagawa H (2009) Burnout and risk factors for arteriosclerotic disease: follow-up study. J Occup Health 51:123–131 19212087 10.1539/joh.l8104

[CR24] Kroenke K, Spitzer RL (2002) The PHQ-9: a new depression diagnostic and severity measure. Psychiatr Ann 32(9):1–7. 10.3928/0048-5713-20020901-06.S2CID17881533

[CR25] Marsland AL, Walsh C, Lockwood K, John-Henderson NA (2017) The effects of acute psychological stress on circulating and stimulated inflammatory markers: a systematic review and meta-analysis. Brain Behav Immun 64:208–219. 10.1016/j.bbi.2017.01.011. (**PMID: 28089638; PMCID: PMC5553449**) 28089638 10.1016/j.bbi.2017.01.011 PMC5553449

[CR26] Maslach C, Leiter MP (1997) The truth about burnout. Jossey-Bass, San Francisco

[CR27] Maslach C, Jackson SE, Leiter MP (1996) Maslach burnout inventory, 3rd edn. Consulting Psychologists Press, Palo Alto, CA

[CR28] Melamed S, Kushnir T, Shirom A (1992) Burnout and risk factors for cardiovascular diseases. Behav Med 18:53–60 1392214 10.1080/08964289.1992.9935172

[CR29] Melamed S, Shirom A, Toker S, Berliner S, Shapira I (2006) Burnout and risk of cardiovascular disease: evidence, possible causal paths, and promising research directions. Psychol Bull 132(3):327–353 16719565 10.1037/0033-2909.132.3.327

[CR30] Niu W, Liu Y, Qi Y, Wu Z, Zhu D, Jin W (2012) Association of interleukin-6 circulating levels with coronary artery disease: a meta-analysis implementing Mendelian randomization approach. Int J Cardiol 157(2):243–252. 10.1016/j.ijcard.2011.12.098. (**PMID: 22261689**) 22261689 10.1016/j.ijcard.2011.12.098

[CR31] O’Connor K, Muller-Neff D, Pitman S (2018) Burnout in mental health professionals: a systematic review and meta-analysis of prevalence and determinants. Eur Psychiatry 53:74–99 29957371 10.1016/j.eurpsy.2018.06.003

[CR32] Orosz A, Federspiel A, Haisch S, Seeher C, Dierks T, Cattapan K (2017) A biological perspective on differences and similarities between burnout and depression. Neurosci Biobehav Rev 73:112–122 27993607 10.1016/j.neubiorev.2016.12.005

[CR33] Papadopoulos A, Palaiopanos K, Björkbacka H, Peters A, de Lemos JA, Seshadri S, Dichgans M, Georgakis MK (2022) Circulating interleukin-6 levels and incident ischemic stroke: a systematic review and meta-analysis of prospective studies. Neurology 98(10):e1002–e1012. 10.1212/WNL.0000000000013274. (**PMID: 34969940; PMCID: PMC8967391**) 34969940 10.1212/WNL.0000000000013274 PMC8967391

[CR34] Pedersen BK, Febbraio MA (2007) Point: interleukin-6 does have a beneficial role in insulin sensitivity and glucose homeostasis. J Appl Physiol 102:814–816 17068210 10.1152/japplphysiol.01208.2006

[CR35] Pedersen BK, Febbraio MA (2008) Muscle as an endocrine organ: focus on muscle-derived interleukin-6. Physiol Rev 88(4):1379–1406. 10.1152/physrev.90100.2007. (**PMID: 18923185**) 18923185 10.1152/physrev.90100.2007

[CR36] Pedersen SS, von Känel R, Tully PJ, Denollet J (2017) Psychosocial perspectives in cardiovascular disease. Eur J Prev Cardiol 24(3_suppl):108–115 28618908 10.1177/2047487317703827

[CR37] Pruessner JC, Kirschbaum C, Meinlschmid G, Hellhammer DH (2003) Two formulas for computation of the area under the curve represent measures of total hormone concentration versus time-dependent change. Psychoneuroendocrinology 28(7):916–931. 10.1016/s0306-4530(02)00108-7. (**PMID: 12892658**) 12892658 10.1016/s0306-4530(02)00108-7

[CR38] Ridker PM, Rane M (2021) Interleukin-6 signaling and anti-interleukin-6 therapeutics in cardiovascular disease. Circ Res 128(11):1728–1746. 10.1161/CIRCRESAHA.121.319077. (**PMID: 33998272**) 33998272 10.1161/CIRCRESAHA.121.319077

[CR39] Ridker PM, Rifai N, Stampfer MJ, Hennekens CH (2000) Plasma concentration of interleukin-6 and the risk of future myocardial infarction among apparently healthy men. Circulation 101(15):1767–1772. 10.1161/01.cir.101.15.1767 10769275 10.1161/01.cir.101.15.1767

[CR40] Rotenstein S, Torre M, Ramos MA, Rosales RC, Guille C, Sen S, Mata DA (2018) Prevalence of burnout among physicians: a systematic review. JAMA 320(11):1131–1150 30326495 10.1001/jama.2018.12777 PMC6233645

[CR41] Schaufeli W, Buunk B (2004) Burnout: an overview of 25 years of research and theorizing. Wiley. 10.1002/0470013400.ch19

[CR42] Scheller J, Chalaris A, Schmidt-Arras D, Rose-John S (2011) The pro- and anti-inflammatory properties of the cytokine interleukin-6. Biochim Biophys Acta 1813(5):878–888. 10.1016/j.bbamcr.2011.01.034. (**PMID: 21296109**) 21296109 10.1016/j.bbamcr.2011.01.034

[CR43] Schnohr P, Marott JL, Kristensen TS, Gyntelberg F, Grønbæk M, Lange P, Jensen MT, Jensen GB, Prescott E (2015) Ranking of psychosocial and traditional risk factors by importance for coronary heart disease: the Copenhagen City heart study. Eur Heart J 36(22):1385–1393 25681607 10.1093/eurheartj/ehv027

[CR44] Shanefelt TD, Hasan O, Dyrbye LN, Sinsky C, Satele D, Sloan J, West CP (2015) Changes in burnout and satisfaction with work-life balance in physicians and the general US working population between 2011 and 2014. Mayo Clin Proc 90(12):1600–1613 26653297 10.1016/j.mayocp.2015.08.023

[CR45] Starkie R, Ostrowski SR, Jauffred S, Febbraio M, Pedersen BK (2003) Exercise and IL-6 infusion inhibit endotoxin-induced TNFalpha production in humans. FASEB J 17:884–886 12626436 10.1096/fj.02-0670fje

[CR46] Tang L, Pang Y, He Y, Chen Z, Leng J (2018) Burnout among early-career oncology professionals and the risk factors. Psychooncology 27(10):2436–2441 30067312 10.1002/pon.4847

[CR47] Toker S, Shirom A, Shapira I, Berliner S, Melamed S (2005) The association between burnout, depression, anxiety, and inflammation biomarkers: C-reactive protein and fibrinogen in men and women. J Occup Health Psychol 10(4):344–362. 10.1037/1076-8998.10.4.344. (**PMID: 16248685**) 16248685 10.1037/1076-8998.10.4.344

[CR48] Toker S, Melamed S, Berliner S, Zeltser D, Shapira I (2012) Burnout and risk of coronary heart disease: a prospective study of 8838 employees. Psychosom Med 74:840–847 23006431 10.1097/PSY.0b013e31826c3174

[CR49] Uciechowski P, Dempke WCM (2020) Interleukin-6: A Masterplayer in the Cytokine Network. Oncology 98(3):131–137. 10.1159/000505099. (**Epub 2020 Jan 20 PMID: 31958792**) 31958792 10.1159/000505099

[CR50] von Känel R (2015) Acute mental stress and hemostasis: When physiology becomes vascular harm. Thromb Res 135(Suppl 1):S52–S55 10.1016/S0049-3848(15)50444-1 PMC4386736 25861135

[CR51] von Känel R, Kudielka BM, Preckel D, Hanebuth D, Fischer JE (2006) Delayed response and lack of habituation in plasma interleukin-6 to acute mental stress in men. Brain Behav Immun 20(1):40–48. 10.1016/j.bbi.2005.03.013. (**PMID: 15890495**) 15890495 10.1016/j.bbi.2005.03.013

[CR52] von Känel R, Bellingrath S, Kudielka BM (2008) Association between burnout and circulating levels of pro- and anti-inflammatory cytokines in schoolteachers. J Psychosom Res 65(1):51–59. 10.1016/j.jpsychores.2008.02.007. (**PMID: 18582612**) 18582612 10.1016/j.jpsychores.2008.02.007

[CR53] Weisberg SP, McCann D, Desai M, Rosenbaum M, Leibel RL, Ferrante AW Jr (2003) Obesity is associated with macrophage accumulation in adipose tissue. J Clin Invest 112:1796–1808 14679176 10.1172/JCI19246 PMC296995

[CR54] Zannas AS, Gordon JL, Hinderliter AL, Girdler SS, Rubinow DR (2020) IL-6 response to psychosocial stress predicts 12-month changes in cardiometabolic biomarkers in perimenopausal women. J Clin Endocrinol Metab 105(10):e3757–e3765. 10.1210/clinem/dgaa476. (**PMID: 32706883; PMCID: PMC7465560**) 32706883 10.1210/clinem/dgaa476 PMC7465560

